# A 3D printed model of the female pelvis for practical education of gynecological pelvic examination

**DOI:** 10.1186/s41205-022-00139-7

**Published:** 2022-05-05

**Authors:** Matthias Kiesel, Inga Beyers, Adam Kalisz, Ralf Joukhadar, Achim Wöckel, Saskia-Laureen Herbert, Carolin Curtaz, Christine Wulff

**Affiliations:** 1 Department of Gynecology, University Hospital Würzburg, Josef-Schneider-Str. 4, 97080 Würzburg, Germany; 2 Institute of Electric Power Systems (IfES), Leibniz Universität, Hannover, Appelstraße 9A, 30167 Hannover, Germany; 3 Department of Electrical, Electronic and Communication Engineering, Information Technology (LIKE), Friedrich-Alexander-Universität Erlangen-Nürnberg, Am Wolfsmantel 33, Erlangen, Germany

**Keywords:** Gynecology, Pelvic examination, Pelvic palpation, 3D printing, FDM, SLA, Teaching, Visualization, Education

## Abstract

**Background:**

Pelvic palpation is a core component of every Gynecologic examination. It requires vigorous training, which is difficult due to its intimate nature, leading to a need of simulation. Up until now, there are mainly models available for mere palpation which do not offer adequate visualization of the concerning anatomical structures. In this study we present a 3D printed model of the female pelvis. It can improve both the practical teaching of gynecological pelvic examination for health care professionals and the spatial understanding of the relevant anatomy.

**Methods:**

We developed a virtual, simplified model showing selected parts of the female pelvis. 3D printing was used to create a physical model.

**Results:**

The life-size 3D printed model has the ability of being physically assembled step by step by its users. Consequently, it improves teaching especially when combining it with commercial phantoms, which are built solely for palpation training. This is achieved by correlating haptic and visual sensations with the resulting feedback received.

**Conclusion:**

The presented 3D printed model of the female pelvis can be of aid for visualizing and teaching pelvic anatomy and examination to medical staff. 3D printing provides the possibility of creating, multiplying, adapting and sharing such data worldwide with little investment of resources. Thus, an important contribution to the international medical community can be made for training this challenging examination.

## Background

The female pelvic anatomy is often underrepresented in regular clinical curricula and we experience a lack of knowledge and understanding by many medical students. The gynecological examination, consisting of the bimanual palpation, is of great importance in everyday clinical practice. For instance, it is one of the first steps for a potential indication for surgery. Moreover, the classification of cervical cancer, and with it the possible therapy-options as well as later on aftercare, is based on the findings of the bimanual, rectovaginal palpation [1]. Consequently, early-on, vigorous training of these technical skills is of great importance.

Furthermore, due to the intimate nature of this examination, it is difficult to justify performing training by utilizing regular clinical patients for teaching medical students. In our opinion, this leads to the need of sufficiently simulating this process, in order to provide the next generation of gynecologists with ample practical training before confronting them with patients.

Currently, there are mainly two types of female pelvic models available. One type is for mere visualization, often made out of rigid plastics and showing various different anatomical structures. The other types of models are made for palpation and consist of closed systems, which can only be explored and utilized by touch. Neither system has been the subject of recent scientific interest for the field of training gynecologic examination. The sparse amount of data is especially critical when taking into account the clinical significance this issue has and the insecurity unprepared medical students feel about this sensitive examination [2–6]. Moreover, there is data showing that medical students generally benefit from simulation and that their confidence rises with the amount of performed examinations, consequently supporting the value of simulation [5–7]. Furthermore, there are findings indicating a beneficial use of specialized models for teaching the gynecological examination. Dilaveri et al. for instance could show in a meta-analysis that the existing studies, although few in number, do show significant benefit for trainees when being able to perform training with a pelvic model [8]. Adding to this, there are studies indicating the usefulness of 3D printing for creating models for different medical specialties, including pelvic anatomy, which can be used for teaching for instance medical students [9–14].

**Fig. 1:**
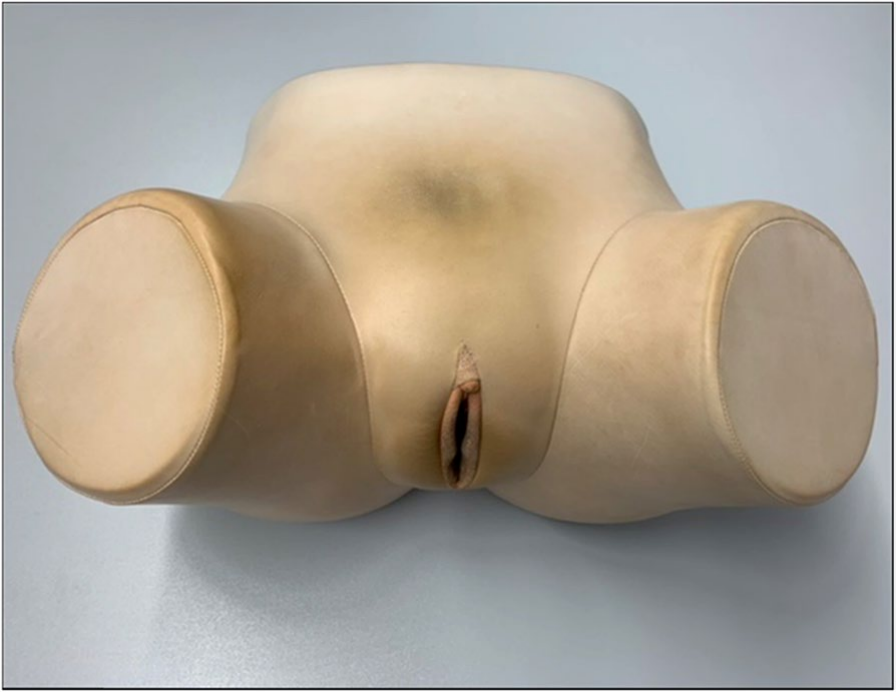
Commercial model of the company *Schultes medacta GmbH & Co Lehrmodelle KG* for training of pelvic examination

At the department of Gynecology of the University Hospital Würzburg, medical students in their fifth to sixth year of university education regularly receive standard curricular education for gynecological examination and the concerning anatomy. This so-called “skills-lab” includes verbal explanation together with the review of relevant 2D images by using a Microsoft PowerPoint presentation as well as the use of commercial models of the company *Schultes medacta GmbH & Co Lehrmodelle KG*, representing the female pelvis. As most of the available commercial models, these do not show any anatomical structures, but only offer the possibility of palpation. One of these models is shown in Fig. 1. In our experience, the imagination of the anatomy by mere palpation without adequate visualization and comprehension poses a big challenge for most medical students and often leads to frustration. In this study we present a self-made 3D printed model of the female pelvis. It shall be used to improve practical teaching of gynecological pelvic examination, e.g. for medical students.

## Methods

### Model concept

The aim was to create an anatomical model that visualizes crucial anatomical structures in a way, which facilitates medical students’ understanding and memorization. Only pelvic structures crucial for teaching the female pelvic examination were included, i.e. bones, lymph nodes, arterial vessels, venous vessels, nerves, and ligaments (Table 1).

**Table 1:** Anatomical structures depicted in the described 3D model of the female pelvis

Bones:	Spine
	Iliac bone
	Parts of ischial bone
	Parts of pubic bone
Lymph nodes:	Pelvic lymph nodes
	Para-aortic lymph nodes
Arterial vessels:	Aorta
	Internal iliac artery
	External iliac artery
	Uterine Artery
Venous vessels:	Inferior vena cava
	Iliac vein
	Internal iliac vein
Nerval structures:	Superior and Inferior Hypogastric Plexus
Ligaments:	Round Ligaments
	Sacro-uterine ligaments
	Parametrium
	Symphysis
	Ovarian Ligament
	Suspensory ligament of ovary (= Infundibulopelvic ligament)
Uterus with Portio	
Fallopian Tubes	
Ovaries	
Pelvic floor	
Urinary tract:	Bladder
	Ureter
	Urethra
Vulva:	Labia minora
	Labia majora
	Clitoris
Vagina	
Rectum	
Anus	

### The virtual model

**Fig. 2:**
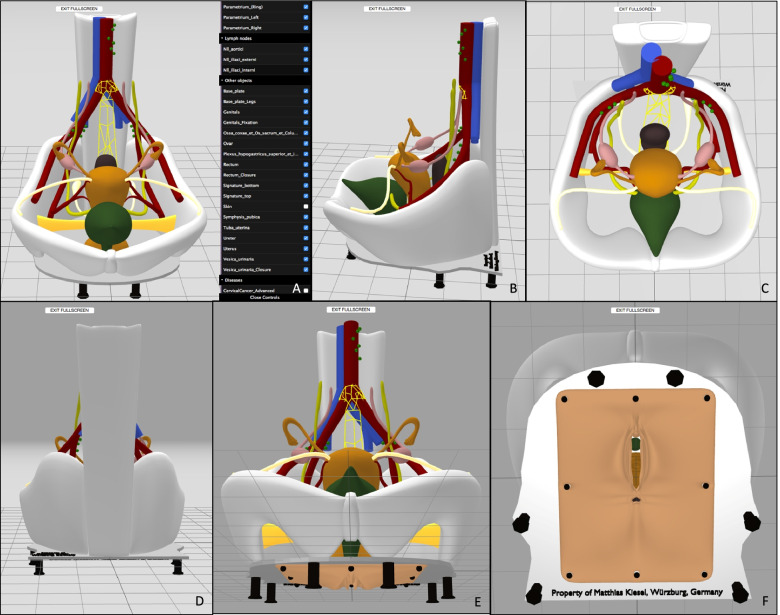
**A**-**F** Pictures showing virtual model of the female pelvis from different angles. **A** The drop-down-menu on the right-hand-side of the screen depicts the different structures, which can be hidden and revealed. *Blender*, version 2.93

**Fig. 3:**
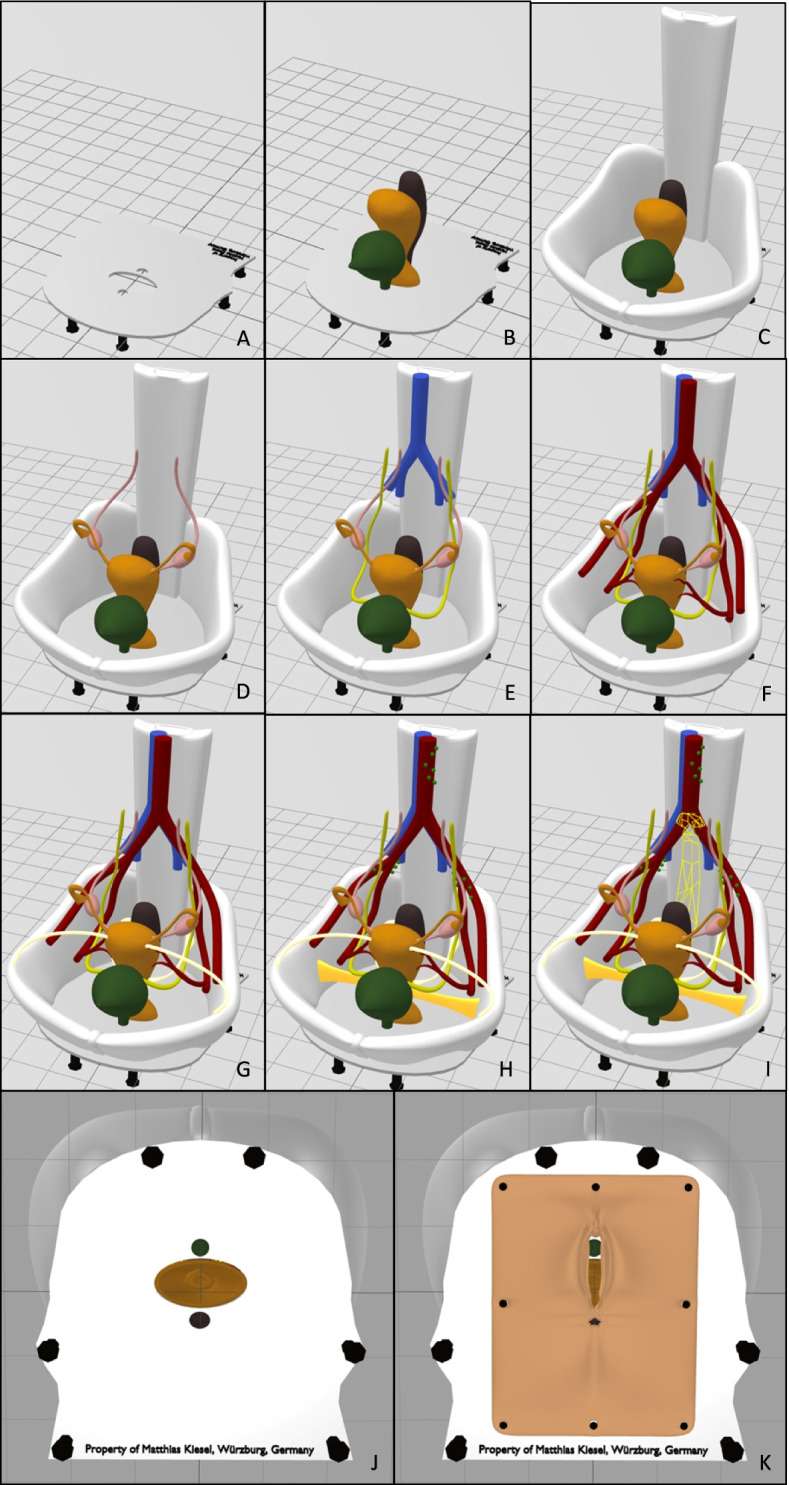
**A**-**K** “Constructing” the model step by step by revealing its separate structures with the program *Blender*, version 2.93. **J** and **K** show the bottom side of the model. **J** bare bottom side. **K** vulva with urethra and anal opening

As a first step, the program Blender, version 2.93 was used, in order to virtually build the simplified model showing selected parts of the female pelvis. Blender is a free and open-source 3D creation suite which is typically used for freeform modeling. The design process of the model is based on illustrations and anatomical data, which were retrieved from the book PROMETHEUS Lernatlas der Anatomie, 2nd edition from Georg Thieme Verlag KG, Taschenbuch Anatomie, 1st edition from Elsevier GmbH as well as on clinical experience. Pictures of the virtual model of the female pelvis can be seen in Fig. 2. The process of “constructing” the model by revealing separate structures is shown in Fig. 3.

**Fig. 4:**
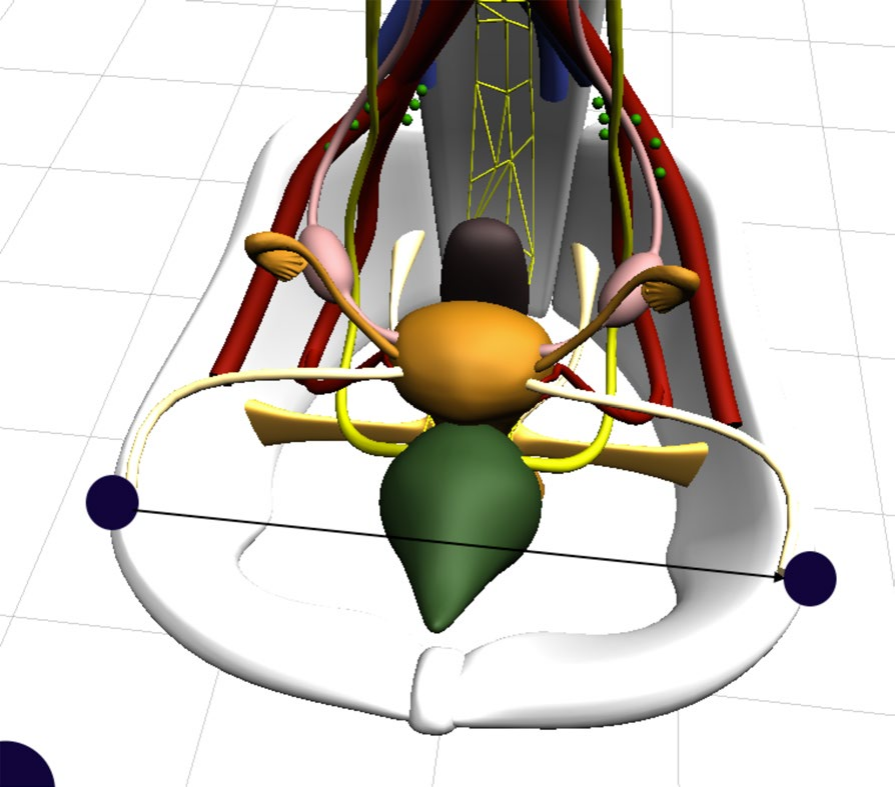
Distancia interspinosa depicted by the black arrow between the two dots, *Blender*, version 2.93

We slightly altered certain parameters for the sake of simplicity, easier accessibility and therefore expectedly improved learning results. On the one hand, the scale of the depth of the pelvic bones was increased. By doing so, we created greater space in the simulated pelvic cavity, enabling us to place the single structures with more space between them. The result was a more comprehensible model at the cost of a slight reduction of its anatomical correctness, as especially the shape of the pelvic bones was simplified and the distance to the uterus, the bladder and the rectum was increased. On the other hand, the width of the pelvis was chosen according to the distance of the anterior iliac spines with 28 cm (Fig. 4). This measure was taken from the existing models from *Schultes medacta GmbH & Co Lehrmodelle KG* to create a sufficient congruence and comparability between the two models.

### The 3D printing process

The nomenclature for the 3D printing manufacturing process used in this work refers to the American Society for Testing and Materials (ASTM) standards [15].

**Fig. 5:**
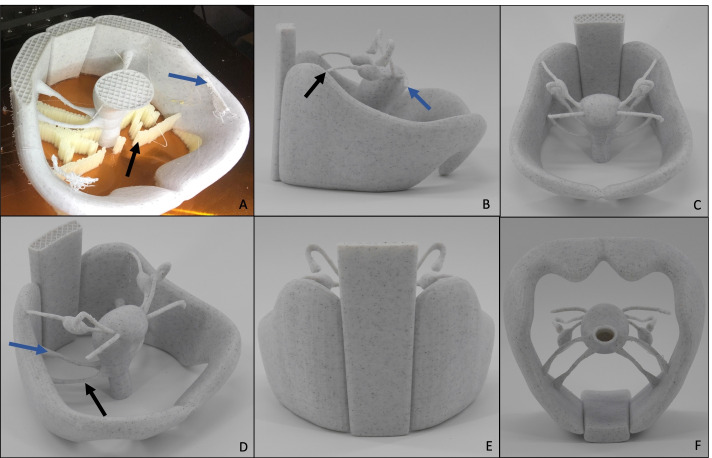
**A**-**F** The first version of the printed model, consisting of only selected parts of the original virtual model: spine, pelvic bones, uterus, ovaries with ligaments, fallopian tube, vagina, parametrium and sacroiliac ligament presented from different angles. **A** Depiction of the printing process. The infill material can be seen, as the inside of the model is not printed of massive PLA. Black arrow: support material. Blue arrow: incorrectly printed material (originally planned to be part of the round ligament). 3D printer: *Craftbot 3*, company: CRAFTUNIQUE, Budapest, Hungary, filament: PLA, color: Marble, filament-diameter: 1,75 mm from the company Geeetech, Shenzhen, China. **B** Black arrow: anatomically incorrect insertion of the suspensory ligament of ovary. This insertion-point was chosen, in order to achieve sufficient stability for the printed model. Blue arrow: insufficiently printed round ligament. Here the strong overhang, leading to the ligament being printed in “midair”, lead to a flaw during the printing process. **D** Black arrow: parametrium. Blue arrow: sacrouterine ligament

**Fig. 6:**
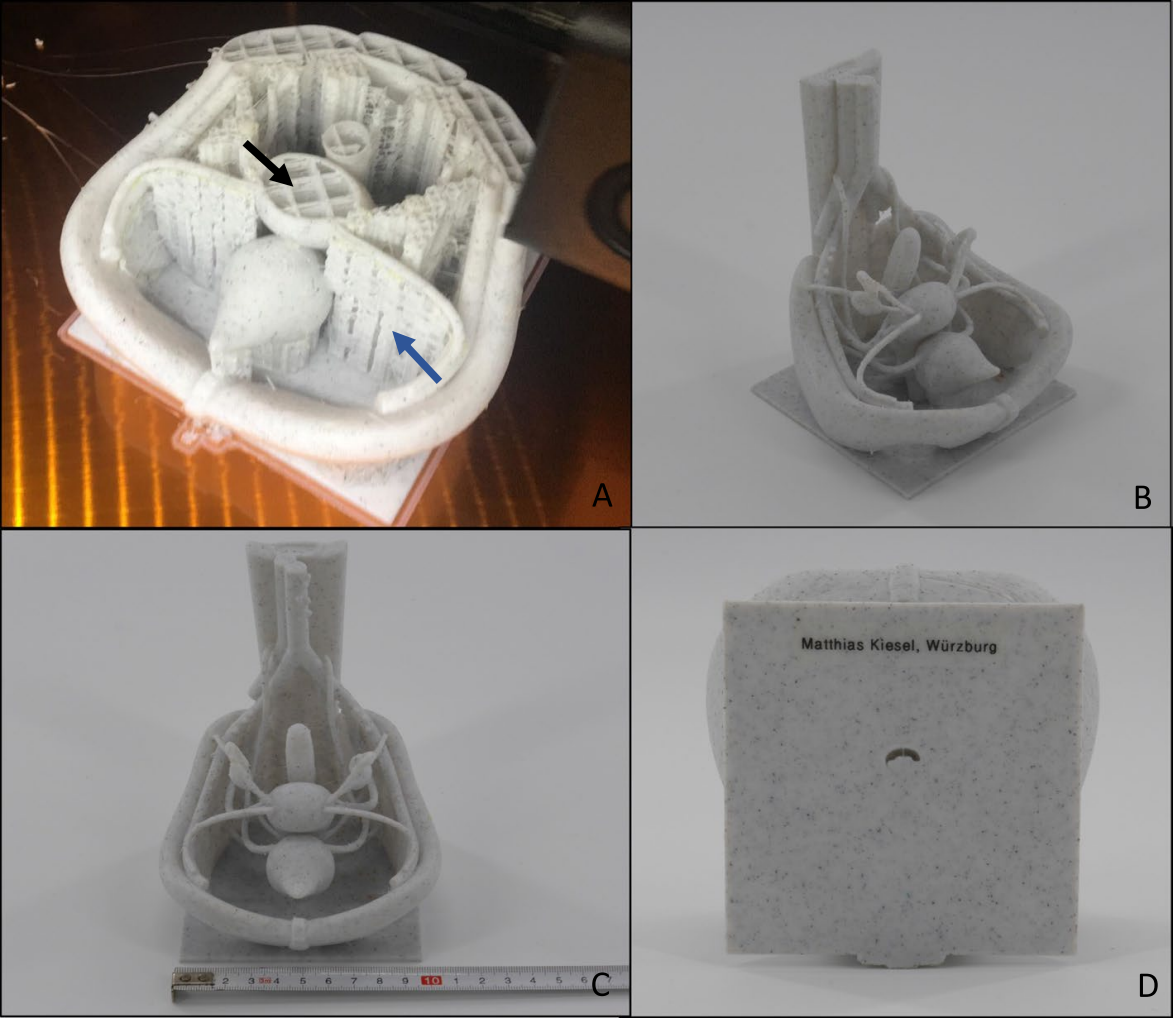
**A**-**D** 3D printed model of the female pelvis, printed in one piece. **A** Process of 3D printing. 3D printer: *Craftbot 3*, company: CRAFTUNIQUE, Budapest, Hungary, filament: PLA, color: Marble, filament-diameter: 1,75 mm from the company Geeetech, Shenzhen, China. Black arrow: relatively low density of infill material can be seen, as the inside of the model is not printed of massive PLA. This accelerates the printing-process and reduces the amount of filament needed at the cost of stability. Blue arrow: support material, which has to be removed manually after the print. **B** Finished model directly after being removed from the 3D printer. Support material still remaining. **C** Measure showing the size of the model (10 cm width). **D** Vulva and openings for urethra, vagina and anus have not yet been printed

A first Draft was printed using desktop material extrusioin (*Craftbot 3*, CRAFTUNIQUE, Budapest, Hungary) with 1.75 mm diameter, marble colored, polylactic acid (PLA) filament (Geeetech, Shenzhen, China). Figure 5 shows the first print of the model, using PLA. Firstly, the main parts of the model were printed as one piece, in order to receive first impressions of how these sections would fit together. Additionally, the entire model with more added parts was printed as a whole. This is shown in Fig. 6. The following structures of the virtual model were added in this printed version: symphysis, arterial and venous vessels, pelvic and paraaortic lymph nodes, rectum and bladder with urethra and both ureters. These results indicated, that another printing technique other than material extrusioin would have to be added.

**Fig. 7:**
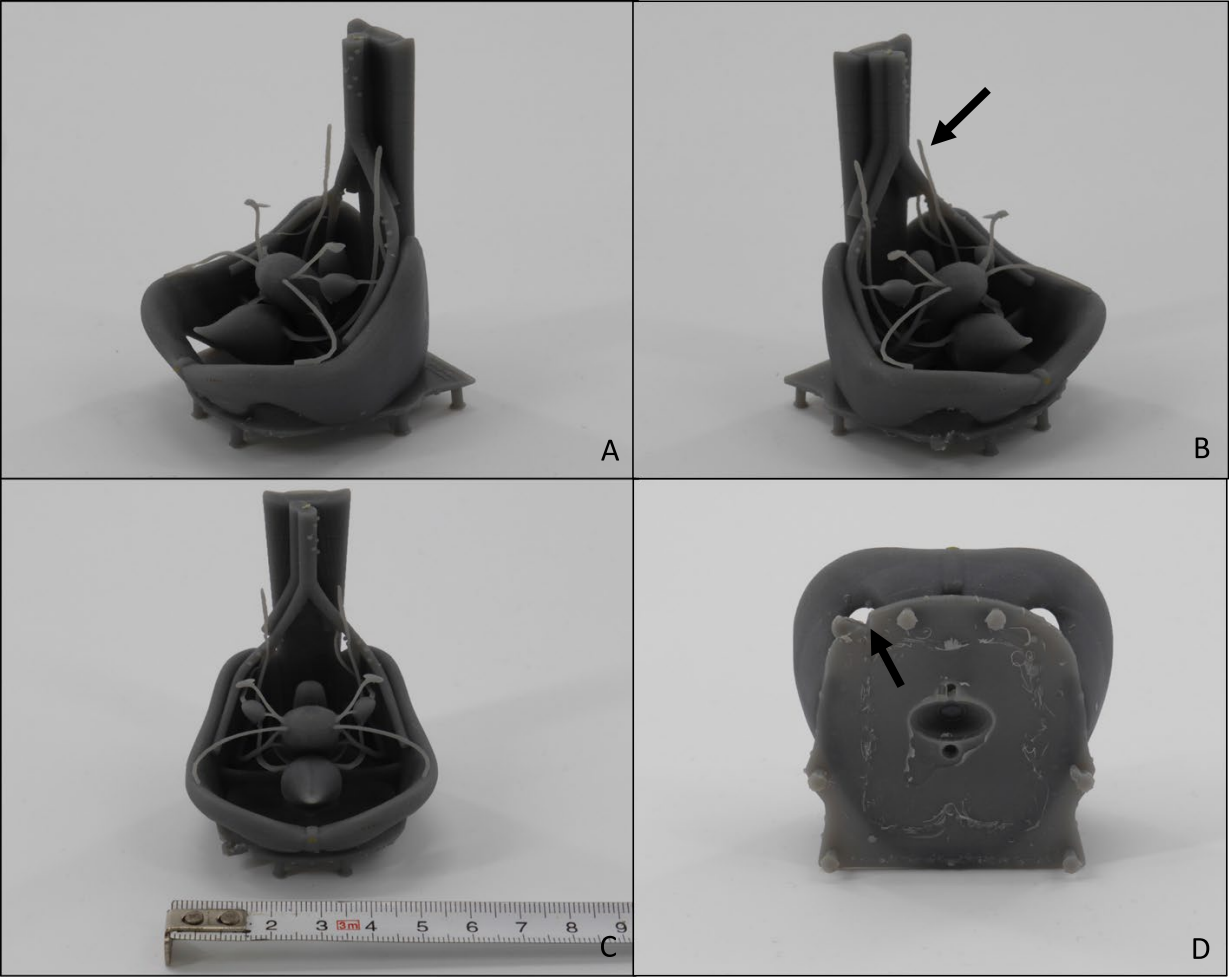
**A**-**D** The third version of the printed model, created by using SLA-printing-technology. Used 3D printer: *SparkMaker Original* from the company SparkMaker, Shenzhen, China. The liquid resin used was *Colored UV Resin* from the company ANYCUBIC, Shenzhen, China. **B** Black arrow: the false insertion of the suspensory ligament of ovary could be corrected, now ending in the air. **C** Because of the relatively small size of the printer’s building area of 98, 6 × 55, 4 mm and building height of 125 mm, this model is relatively small. **D** Black arrows show a flaw in the print of the base plate. As the print of the vulva failed, one can directly see the urethral, vaginal and anal orifice in the base plate

Consequently, for the next iteration of the pelvic model, instead of material extrusioin, a vat photopolymerization technique, desktop stereolithography (SLA) was used (*SparkMaker Original*, Article number: SKUS01, SparkMaker, Shenzhen, China) with a gray colored UV Resin (ANYCUBIC, Shenzhen, China). The liquid resin used was *Colored UV Resin* (grey) (ANYCUBIC*,* Shenzhen, China). Because of the relatively small size of the printer’s building platform of 98, 6 × 55, 4 mm and building area height of 125 mm, this model is relatively small. Nevertheless, the technique of SLA offered the possibility of printing almost the entire model in one single print. The results are displayed in Fig. 7. One can see the improved details, fine structures and less visible layers compared to the former prints. Unfortunately, printers using the SLA-technology and additionally offering an adequately large building area and building height for printing the model e.g. in realistic size, require relatively high financial investments. Furthermore, a model of the female pelvis should be created, which could be assembled step by step by the students. This made a print of the entire model in one pass obsolete.

For the final iteration, a combination of material extrusioin and STL was used. For material extrusioin the *Ultimaker 2+* (Ultimaker BV, Utrecht, Netherlands) and the *Sidewinder X1* (Artillery, Shenzhen, China) were utilized*.* The used filament was PLA (Article number: F10201, DAS FILAMENT, Emskirchen, Germany) as well as PETG (polyethylene terephthalate glycol-modified, Article number: F10174, Emskirchen, Germany) with a filament diameter of 2,85 mm and the color white. PETG has partly similar characteristics as PLA, making no relevant differences in the presented results. For SLA, the printer *Form 3* (Article number: PKG-F3-SVC, Formlabs, Somerville, USA) was used. The resin was *Grey Resin* (Article number: RS-F2-GPGR-04, Formlabs, Somerville, USA).

After SLA-printing, the resin has to be removed, using Isopropanol, from the printed part and the part itself has to be hardened. Therefore, the remaining resin was removed from the parts using Form *Wash* and *Form Cure* (Article number: PKG-F3-SVC, Formlabs, Somerville, USA).

**Fig. 8:**
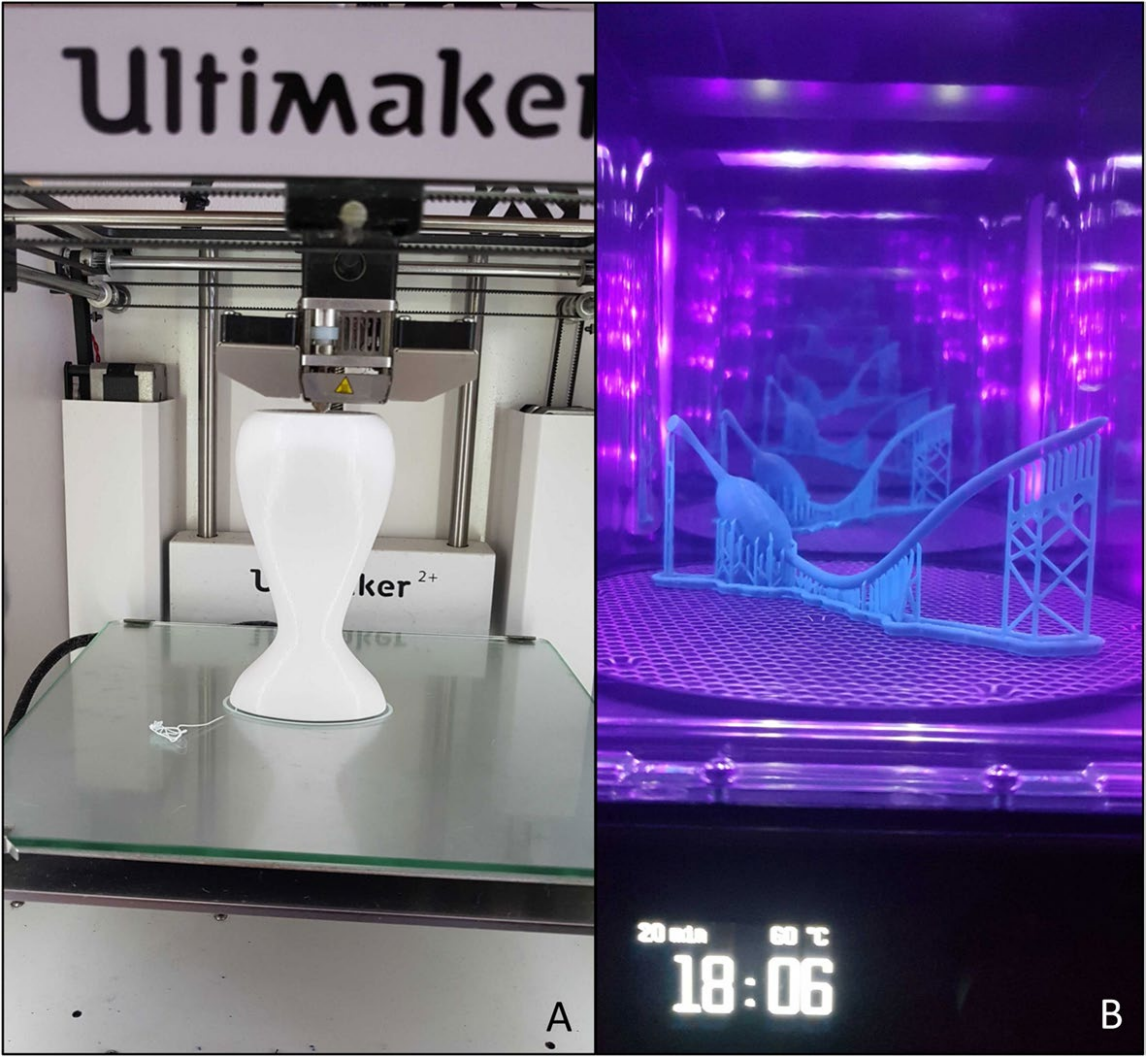
**A**–**B**, **A** Material extrusioin: printing-process of the uterus (*Ultimaker 2+* from the company Ultimaker BV, Utrecht, Netherlands, resin: PLA, color: white, (Article number: F10201) from DAS FILAMENT, Emskirchen, Germany). **B** Curing-process of one ovary with attached filaments after being SLA-printed and after removing the leftover resin. Support-structures still can be seen (*Form Cure* (Article number: PKG-F3-SVC) from Formlabs GmbH, Somerville, USA), resin: *Grey Resin* (Article number: RS-F2-GPGR-04) from Formlabs GmbH, Somerville, USA)

**Fig. 9:**
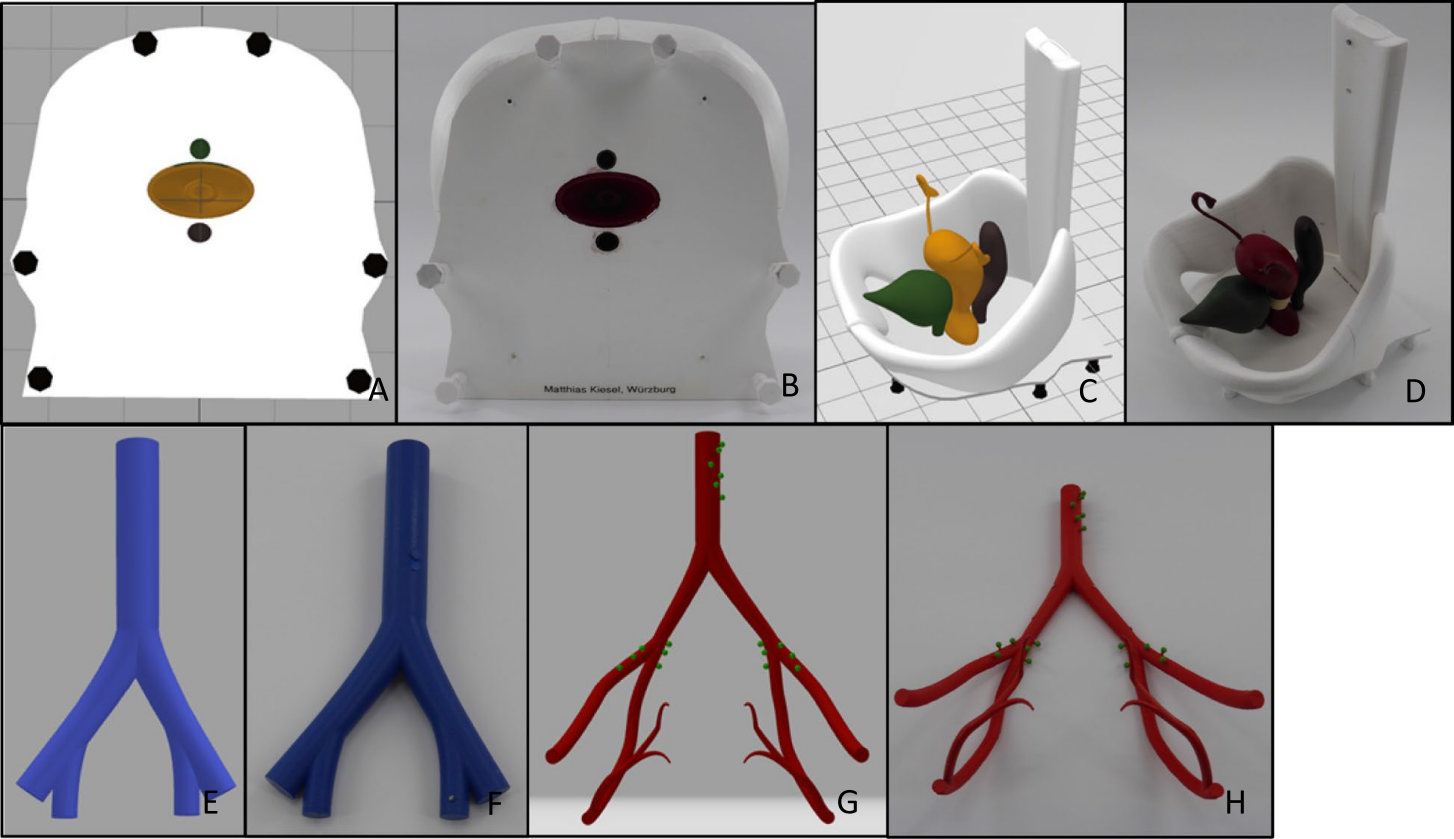
**A**-**H** Pictures of anatomical structures of the pelvic model presented as virtual and printed parts. **A**-**B** Base plate with legs and partly visual urethra, rectum and cervix. **C**-**D** Base plate with legs, pelvic bones, symphysis, bladder with urethra, rectum, uterus and fallopian tubes. **E**-**F** venous vessels. **G**-**H** arterial vessels with lymph nodes

**Fig. 10:**
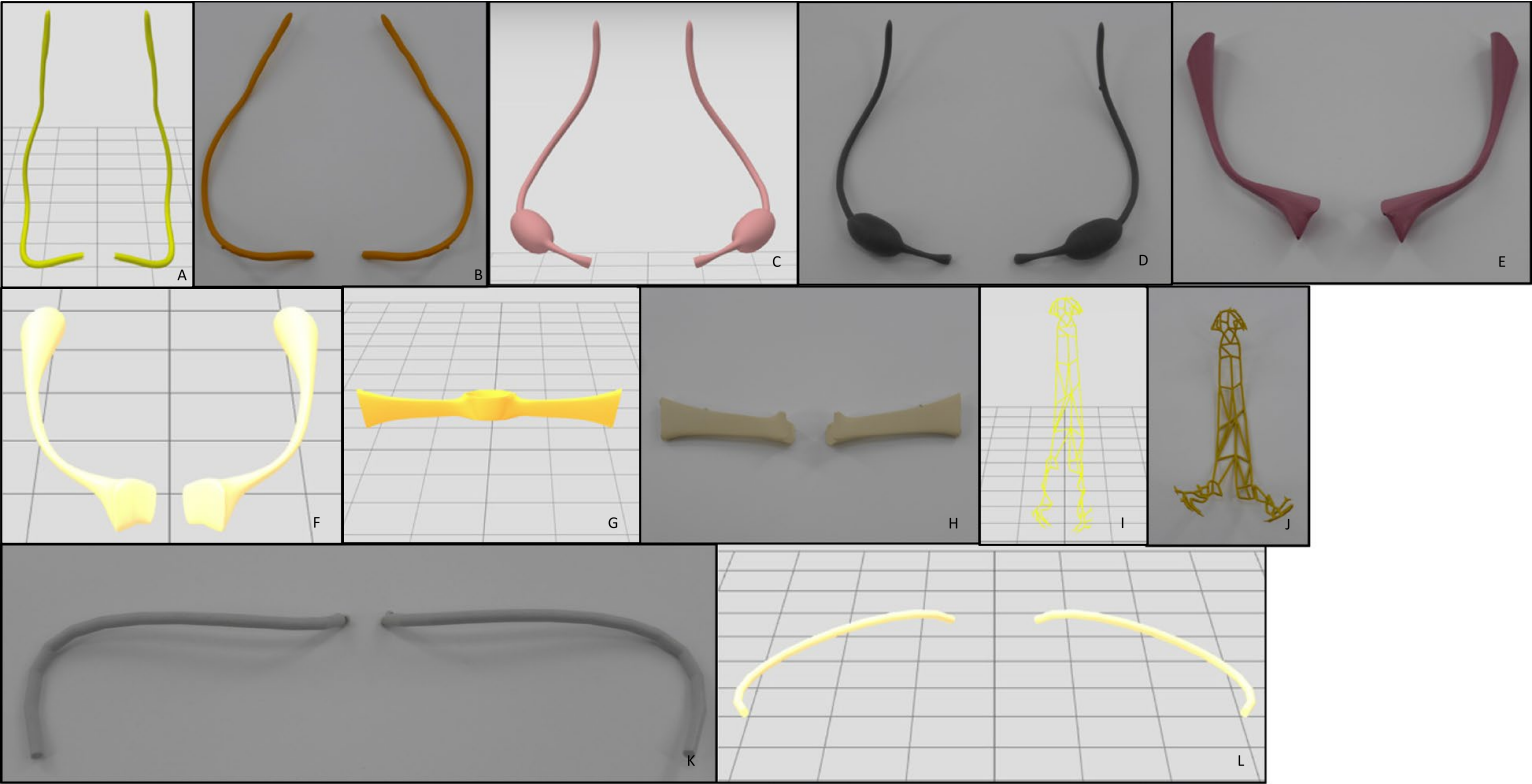
**A**-**L** Pictures of anatomical structures of the pelvic model presented as virtual and printed parts. **A**-**B** ureters, **C**-**D** ovaries with ligaments. **E**-**F** sarcrouterine ligament. **G**-**H** parametrium. **I**-**J** superior and inferior hypogastric plexus. **K**-**L** round ligament

Having prepared the stl-files, they could be transformed into gcodes and the printing process could begin. The slicing for the material extrusioin was done with the open-source slicing software *Cura Version 4.8*. The Stereolithography (SLA) prints were prepared with *PreForm Version 3.14.0*, a proprietary software by Formlabs, Somerville, USA. Pictures of the printing-process can be seen in Fig. 8. Table 2 shows the parts of the model, which were printed using material extrusioin and the ones, which were printed using SLA. In Figs. 9 and 10 one can see a visual comparison of the single anatomical structures in their virtual, 3D printed and colored version. Before painting the parts, a first coating using the product *edding 5200 Permanent Spray Kunsttoffgrundierung, farblos* (Article number: B00CSLMC0U, Edding, Ahrensburg, Germany, purchased via the company Amazon Europe Core S.à r.l., Seattle, USA) was applied. The coloring was done using spray paint (Edding, Ahrensburg, Germany) or manual painting with a brush using colors from the company Revell Gmbh*.* Clear varnish was applied for coating the colored parts by using the product *edding 5200 Permanent Spray Klarlack, seidenmatt* (Article number: B00CSLM46C, Edding, Ahrensburg, Germany, purchased via the company Amazon Europe Core S.à r.l., Seattle, USA). Table 3 lists all colors, which were used and the anatomical structures they were applied to.

**Table 2:** Anatomical structures being printed using material extrusioin and SLA respectively

Parts being printed using FDM:	Bones:	Spine
		Iliac bone
		Parts of ischial bone
		Parts of pubic bone
	Lymph nodes:	Pelvic lymph nodes
		Para-aortic lymph nodes
	Arterial vessels:	Aorta
		Internal Iliac Artery
		External Iliac Artery
	Venous vessels:	Inferior Vena Cava
		Iliac Vein
		Internal Iliac vein
	Ligaments:	Parametrium
		Symphysis
	Uterus with Cervix	
	Pelvic floor	
	Urinary tract:	Bladder
		Urethra
	Vulva:	Labia minora
		Labia majora
		Clitoris
	Vagina	
	Rectum	
	Anus	
Parts being printed using SLA:	Arterial vessels:	Uterine Artery
	Nerval structures:	Superior Hypogastric Plexus
		Inferior Hypogastric Plexus
	Ligaments:	Round Ligaments
		Sacro-uterine Ligaments
		Ovarian Ligament
		Suspensory Ligament of Ovary (= Infundibulopelvic Ligament)
	Fallopian Tubes	
	Ovaries	
	Urinary tract:	Ureters

**Table 3:** Colors, which were used and the anatomical structures they were applied to. Except for the bladder, all parts were colored with spray paint form the company edding International GmbH***.*** The Bladder was manually painted with a brush using the color *Revell, Email-Farbe, dunkelgrün, matt* from the company Revell GmbH (Article number: B0002HZ2W2, purchased via the company Amazon Europe Core S.à r.l., Seattle, USA)

Color	Anatomical structure	Article number
White (Verkehrsweiß)	Spine	B00CSLLQXO
	Iliac bone	
	Parts of ischial bone	
	Parts of pubic bone	
	Symphysis	
	Pelvic floor (Base plate)	
Yellow-green (Gelb-grün)	Pelvic lymph nodes	B00CSLLX5A
	Para-aortic lymph nodes	
Light red (Verkehrsrot)	Aorta	B00CSLL2XS
	Internal Iliac Artery	
	External Iliac Artery	
	Uterine Artery	
Gentina-blue (Enzian-blau)	Inferior Vena Cava	B00CSLL3N2
	Iliac Vein	
	Internal Iliac Vein	
Light yellow (Verkehrsgelb)	Superior Hypogastric Plexus	B00CSLL63O
	Inferior Hypogastric Plexus	
	Round Ligaments	
Mauve (Hellviolett)	Sacro-uterine Ligaments	B06XNMSGQV
Ivory (Elfenbein)	Parametrium	B00CSLLNOG
No separate painting	Ovarian Ligament	
	Suspensory Ligament of Ovary (= Infundibulopelvic Ligament)	
	Ovaries	
Dark green (Dunkelgrün)	Bladder	*Revell GmbH*, B0002HZ2W2
	Urethra	
Sunny yellow (Sonnengelb)	Ureter	B00CJ3OWUU
Powdery (Puder Pfirsich)	Labia Minora	B087FQHGXW
	Labia Majora	
	Clitoris	
Light grey (Lichtgrau)	Round Ligament	B00CSLLUVC
Chocolate-brown (Schokoladen-braun)	Rectum	B00CSLL82I
Dark red (Purpurrot)	Uterus with Cervix	B00CSLLD50

### Mechanical assembly

**Fig. 11:**
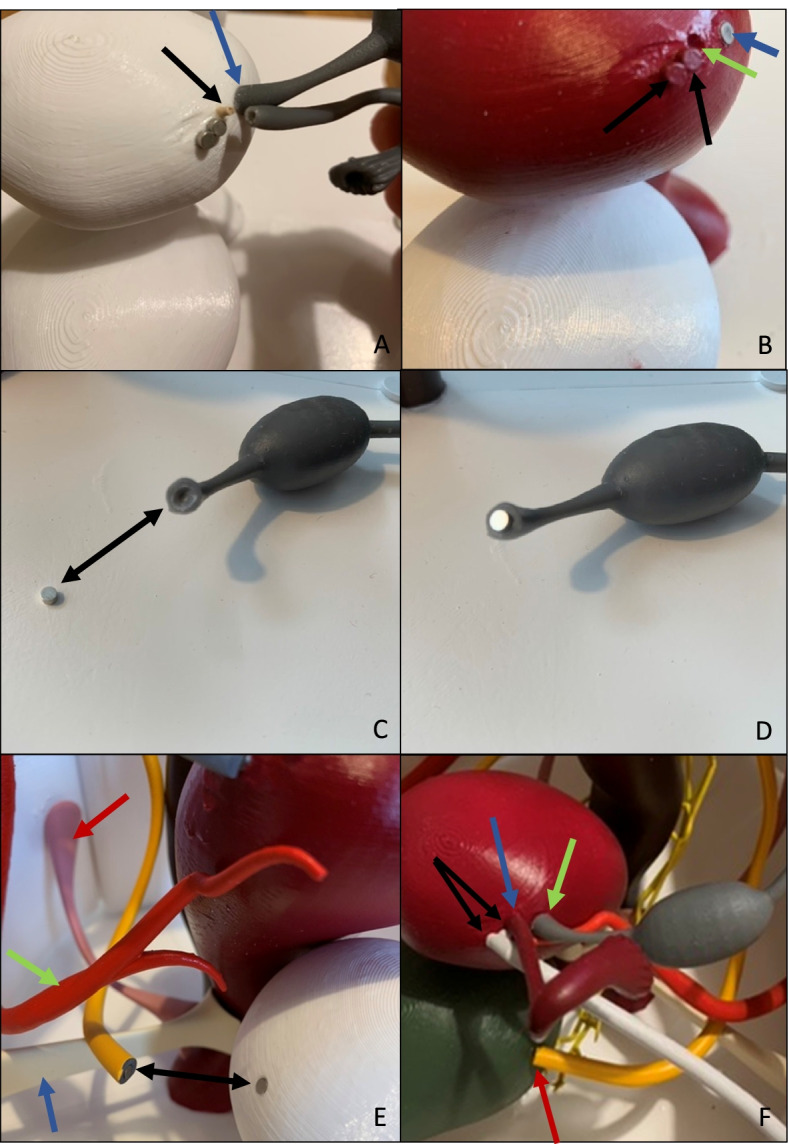
**A**-**F** Different attempts of connecting single parts of the model. **A** Black arrow: First attempts of connecting the fallopian tube to the uterus by drilling a whole in both parts and connecting them with a wooden stick. Blue arrow: The ovarian ligament was glued to the uterus in a first attempt using super glue (*Pattex Sekundenkleber Ultra Gel* from the company Henkel AG & Co. KGaA, Düsseldorf-Holthausen, Germany). **B** Two black arrows: The insertion of the round ligament into to uterus was created by gluing two small magnets onto the uterus and the round Ligament. With two magnets, the round ligament could be held in position. Green arrow: hole for connection-piece for fallopian tube. Blue arrow: similar to the round ligament, we used a magnet for the ovarian ligament. Yet we drilled a hole in the uterus and the ovarian ligament, in which we inserted the two magnets. This resulted in an optically more fluid transition between the two connected objects. **C**-**D** Magnet for connecting ovarian ligament to uterus. **E** Black double-arrow: ureters and bladder were equipped with small inserted magnets for connection. Blue arrow: parametrium. Green arrow: uterine artery. Red arrow: sacrouterine ligament. **F** Black arrows: two small magnets were glued onto the uterus for connecting the round ligament. Blue arrow: The fallopian tube was glued to the uterus with super glue (*Pattex Sekundenkleber Ultra Gel* from the company Henkel AG & Co. KGaA, Düsseldorf-Holthausen, Germany). Green Arrow: The ovarian ligament was connected to the uterus by using inserted magnets. Red arrow: The ureters were connected to the bladder by using inserted magnets. The magnets were from the company Brudazon UG, Hamm, Germany, (Article number: B07L22SJZR, purchased via the company Amazon Europe Core S.à r.l., Seattle, USA)

To ensure the model can be assembled by students, the individual organs need to have mechanical fastening mechanisms that allow easy assembly, but do not pose a distraction from the actual anatomical model. There are a number of options, which are shown in Table 4. For the first version of the pelvic model that could be assembled in parts, a combination of magnetic inserts (Brudazon UG, Article number: B07L22SJZR, Hamm, Germany, purchased via the company Amazon Europe Core S.à r.l., Seattle, USA) and gluing (*Pattex Sekundenkleber Ultra Gel* (Henkel AG & Co. KGaA*,* Düsseldorf-Holthausen, Germany)) was used. However, this did not prove ideal, as magnets are unsuited to prevent rotational movement and gluing reduced the portion of the model that can be assembled. The subsequent version relies on a combination of inserted magnets, press-fits and metal nuts and screws. This allows for a high amount of manual assembly by students, while each fastening is optimized for the respective materials and the relative position of the parts. As the phantom should additionally be able to simulate the position a patient has when being seated on a gynecological chair, 6 small columns were added to the model’s base plate, which raised it several centimeters. This led to the Vulva being elevated from the ground, now remaining in midair. When tilting the entire model around 90°, laying it on the spine, one can simulate the patient’s posture during gynecological examination. The extra wide rear end of the base plate provides ample stabilization in this position. Figure 11 displays different attempts of connecting single parts of the model.

**Table 4:** Options for fastening mechanisms for assembling the 3D printed model

	Permanent	Non-permanent
Purely 3D printed	• Friction Welding	• Press-fit • 3D printed threads and screws/bolts
Additional Material/Components needed	• Gluing • Solvent welding	• Metal nuts and screws/bolts • Heat-inserted metal threads and screws/bolts • Magnets

## Results

**Fig. 12:**
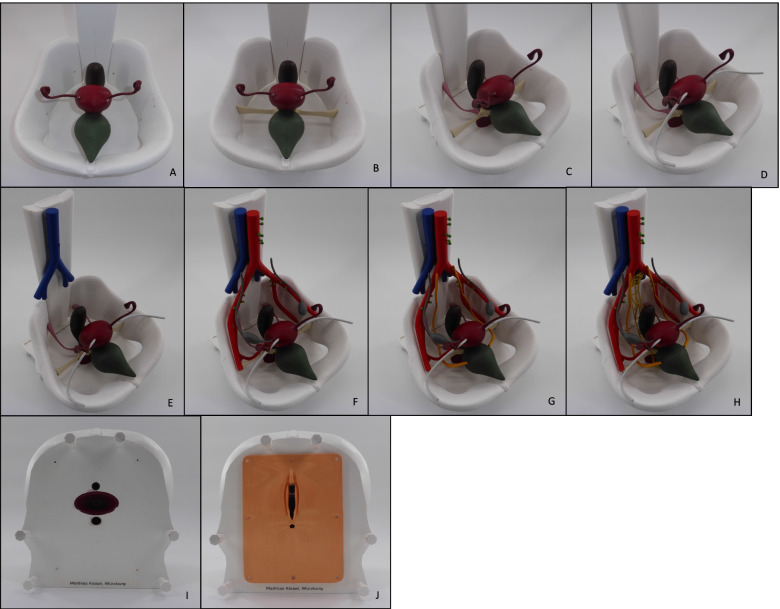
**A**–**J** The process of manually “constructing” the 3D printed model of the female pelvis. **A** Base plate (pelvic floor) together with bladder and urethra, uterus with vagina, rectum and pelvic bones. **B** parametrium. **C** sacrouterine ligament. **D** round ligament. **E** venous vessels (inferior vena cava, iliac vein, internal iliac vein). **F** arterial vessels (aorta, internal iliac artery, external iliac artery, uterine artery) with lymph nodes (pelvic and paraaortic) and ovaries with ovarian ligament and suspensory ligament of ovary (= infundibulopelvic ligament). **G** ureters. **H** superior and inferior hypogastric plexus. **I** bare base plate (pelvic floor) with urethral, vaginal and anal orifice. **J** vulva with labia majora and minora, clitoris and anus

We present a self-made, life-size model of the female pelvis, created by 3D printing. It can be used for medical students’ practical teaching of gynecological pelvic examination and supports the visualization of anatomical structures important for palpation in this context. Adding to the basic anatomical knowledge learned from lectures and books, it can serve the better understanding of the pelvic anatomy in three-dimensional space. We believe that its utility can be increased when being combined with commercial phantoms, which are built for mere palpation training due to the correlation of haptic and visual sensations and the resulting feedback received by this. The reduction of the components, emphasizing the crucial anatomical structures, helps create an overview as well as supporting swift comprehension. The possibility of assembling the single anatomical structures helps the students to be more actively involved into the teaching, again prompting better learning effects. Thanks to the model being relatively simply structured and the possibility of further reducing momentarily unnecessary parts for a certain explanation, the model can also be used for teaching other medical staff in general or for patient education, such as explaining steps of gynecological surgery. By using 3D printing technology, this model can be created, multiplied, adapted and shared worldwide with little investment of resources. Pictures documenting the process of manually assembling the model are shown in Fig. 12.

The costs depend on the available 3D printers and the specific material choice, but it is expected that the total material costs plus printing costs of the complete model do not exceed 200 EUR. The print preparation and post-processing and assembly are estimated at 10 h, in large parts due to the extensive post-processing requirements of SLA prints. An overview of the costs associated with version 2 of the pelvic model can be found in Tables 5, 6, and 7.

**Table 5:** Summary of required resources for production of model

Part	Number of Prints	Printing Method	Material requirement per part^a^	Total Material Cost in EUR	Total Printing Time in hours^b^	Printer	Printing Cost in EUR
Ossa Coxae et Os Sacrum et Columna Vertebralis + Symphysis Pubica	1	FDM	580 g PLA	14.50	50	AS X1	15.50
Uterus	1	FDM	80 g TPU	6.40	14	AS X1	4.34
Rectum	1	FDM	35 g TPU	2.80	4	AS X1	1.24
Vesica Urinaria	1	FDM	75 g TPU	6.00	12	AS X1	3.72
Genitals	1	FDM	115 g TPU	9.20	20	AS X1	6.20
Ovar	2	SLA	16 ml Resin	7.36	4	Form 3	3.28
Ligamentum Rotundum (Teres Uteri)	2	SLA	7 ml Resin	3.22	3.5	Form 3	2.87
Tuba uterina	2	SLA	7 ml Resin	3.22	3	Form 3	2.46
Venous vessels	1	FDM	45 g PLA	1.13	4.5	UM2+	2.03
Arterial vessels	1	FDM	115 g PLA	2.88	21.5	AS X1	6.67
Ligamentum Sacrouterinum	2	SLA	11 ml Resin	5.06	2	Form 3	1.64
Parametrium	2	SLA	10 ml Resin	4.60	1.5	Form 3	1.23
Ureter	2	SLA	12 ml Resin	5.52	8	Form 3	6.56
Plexus Hypogastricus Superior et Inferior	1	SLA	27 ml Resin	6.21	9	Form 3	7.38
Base Plate	1	FDM	140 g PLA	3.50	14	AS X1	4.34
				81.59 EUR	171 h		65.11 EUR

^a^Rounded up to 5 g / 1 ml

^b^Rounded up to the nearest half hour

**Table 6:** Parameters for production costs

Material Costs	PLA	0.03 EUR/gr
	TPU/TPE	0.08 EUR/gr
	Grey Resin	0.23 EUR/ml
Printing Costs (Amortisation + Electricity)	Ultimaker 2+	0.45 EUR/h
	Form 3	0.82 EUR/h
	Artillery Sidewinder X1	0.31 EUR/h

**Table 7:** Further Material and Time Invest

Neodym Magnets Ø = 10 mm, 2 mm height	16 x	2.72 EUR
Neodym Magnets Ø = 3 mm, 2 mm height	8 x	1.90 EUR
Superglue	3 g	4.00 EUR
Screws M4 with Nuts	4 x	0.50 EUR
Isopronanol	5 l	18.00 EUR
Post-Processing and Assembly	10 h	

## Discussion

Our presented model of the female pelvis can aid students and medical staff, as well as patients, to better understand crucial parts of the anatomy of the female pelvis. The potential of model-based simulation has been indicated, for example, by Takestraw et al. in 1985: three groups of medical students (*N* = 17 to 18 in each group) were taught for pelvic examination procedure. Group A did not use any model at all. Group B only observed a demonstration of an anthropomorphic pelvic model for pelvic examination. Group C observed a demonstration and actively practiced with the pelvic model. Afterwards, a team of patient instructors evaluated the pelvic examination the students performed. Additionally, the students evaluated their training themselves. The students who were able to practice with the pelvic model performed better and rated their own learning success higher than those students who had no access to the model [16]. In our opinion, these findings are promising and support the use of pelvic models for the education of medical staff, although they are relatively outdated and contain rather small case numbers. Consequently, new studies are required.

A promising approach of simulation can also be seen by cooperating with specially trained patients. Wånggren et al. could demonstrate in several studies, that such professionally prepared patients, who can give feedback to the examiner during the pelvic examination, can provide good teaching results for medical students [17–19]. The CEAT-study (*Cost-effective analysis of teaching pelvic examination skills using Gynaecology Teaching Associates (GTAs) compared with manikin models*) compared such trained patients to models created for teaching the gynecological palpation of the pelvis. The Gynecology Teaching Associates, interacting with the students examining them and giving feedback, showed a superior teaching effect for medical students compared to pure models [20]. Comparable findings are described in the meta-analysis of Dilaveri et al. [8]. For instance, Johnson et al. compared two classes of medical students in 1975. One class was taught pelvic examination using a GTA, the other used a plastic model. It was shown that the GTA-class improved better than the class using the plastic model [21]. Nelson et al. could also prove the superior training-effects of GTAs when compared to teaching with pelvic models alone in 1978 [22]. Furthermore, Holzmann et al. could show better learning effects for students, when using a GTA or a model compared to pure teaching without GTA or model in 1977 [23]. Yet it has to be noted, that the stated studies are relatively old with the work of Johnson et al. ranging back more than forty years [8]. Consequently, the techniques used for constructing such teaching-models certainly were different, as for example 3D printing was not available. Although teaching with GTAs seems to be superior to teaching with models, GTAs are more expensive than most models and their use for training more difficult to organize. In addition, the use of professionally trained patients is not established in many countries. In order to make teaching effective, the used methods ought to be easily available, quickly installed and not too expensive. This can make a system-wide introduction of the training-approach with GTAs rather difficult.

Another promising approach appears to be the use of electrically enhanced pelvic models, which can give feedback via pressure sensors. Pugh et al. could demonstrate, that these models support medical students with better training than mere lectures without any practical training [24]. In addition, the electrically enhanced models could also show to be superior to regular models for pelvic palpation concerning their training effect [8, 25], although the higher costs of such models must be taken into account. We could not find studies evaluating specially constructed models with the focus of visualization for additionally assisting the use of existing models for teaching pelvic examination.

Beyond the benefits discussed in the literature, our described model presents additional advantages. Firstly, assembling the pelvic model from ground up allows students to interact with the model more intensively than with a static, pre-assembled model. It aims to increase student engagement, due to the active “hands-on” nature of the activity, and to facilitate a deeper understanding and better retention of the coursework. Secondly, 3D printing has both decentralized and accelerated manufacturing processes. It is the method of choice for prototyping, due to its low costs and the possibility of quick, successive iterations of a design. It has enabled low-cost desktop manufacturing, independent of costly workshop infrastructure. 3D printing also allows an independence from global supply chains and shipping routes. An idea can be shared instantly via the Internet and printed in multiple locations worldwide. The benefit of this was demonstrated by the 3D printing community’s response to medical protective equipment supply shortages during the COVID-19 pandemic [26, 27].

In future versions, it is planned to forego the painting process by acquiring PLA in all colors needed, as the manual painting process is cumbersome and should be avoided. Additionally, the use of flexible filament, so-called TPU (thermoplastic polyurethane) will be investigated to better mimic the elasticity of ligaments and organs.

Anatomical models produced by conventional publishers are often expensive and thus inaccessible to less well-funded institutions. By contrast, the 3D printed pelvic model can be produced at low costs by anyone with access to a 3D printer, following the current trend in computer science to distribute creations using open source licenses. Ideally, the model is printed with a combination of an SLA printer for more delicate parts and a material extrusioin printer with a large build volume and a direct drive for printing flexible material. However, this is not a strict requirement. Hence, production costs can be adapted according to the institution’s budget and the available 3D printers. This combination of almost ubiquitous and swift accessibility together with low resource requirements enables education and training of gynecological knowledge and skills to be spread more effectively among health care providers.

### Curriculum integration

The printed model is currently integrated into the already existing weekly skills-lab of the University Clinic Würzburg, which has been described in the background-section of this work. Students in their fifth to sixth year of university education attend a 1,5 h lasting training-course for gynecological examination. The skills-lab starts with the physician explaining anatomy, physiology and basic information required for performing gynecologic examination via Microsoft PowerPoint. After this theoretical introduction, the hands-on-part begins. The novel 3D printed model is presented to the students in its single disassembled parts stored in a box. Every student-group, consisting of 4 students, is asked to assemble the model. Consequently, the theoretical knowledge of the prior lecture can be applied practically. During this period of the skills-lab, the physician assists the student-groups by assembling the model and answers questions. As soon as every group assembled their 3D printed model, the commercial models of the company *Schultes medacta GmbH & Co Lehrmodelle KG* are used. Except for Vulva, Vagina and Cervix, these models’ internal anatomy can only be examined by palpation. The students then begin training the inspection of the female genitalia, the use of different specula, the performance of PAP-smear and the bimanual, rectovaginal palpation. While doing so, each group can continually correlate and compare their findings between the 3D printed and the commercial models. The skills-lab ends with the students assessing the skills-lab by answering an evaluation-form.

### Model assessment

In the future, the 3D printed model will be assessed during the above described skills-lab by randomizing two different student groups (Group A and Group B). Group A will receive training without the 3D printed model. These students will listen to the theoretical introduction at the begin of the skills-lab and then directly start their practical training by working with the commercial models of the company *Schultes medacta GmbH & Co Lehrmodelle KG*. They wont see or assemble the 3D printed model. Group B will additionally experience the 3D printed model as described in the section above. Both groups will assess the skills-lab by using the existing evaluation-form. Moreover, group A and group B will both be asked to answer the same multiple-choice questions focusing on pelvic anatomy, pathophysiology and examination, in order to test the knowledge they acquired during the skills-lab. Furthermore, group B will answer a special evaluation-form, which will enable the students to specifically assess the 3D printed model and its benefits during the skills-lab. By doing so, it is planned to compare both groups concerning their subjective evaluation of the skills-lab itself with or without the 3D printed model as well as their objectively obtained knowledge. Consequently, possibly improved education and teaching results, enabled by the use of the 3D printed model, will be monitored. As to prevent any disadvantage for students, who were randomized to group A, every group-A-student will be allowed to see, assemble and work with the 3D printed model after finishing his/her evaluation of the skills-lab.

## Conclusion

The presented 3D printed model of the female pelvis can be of aid for visualizing and teaching pelvic anatomy and pelvic examination to medical staff such as medical students, especially when combining it with existing models for palpation. 3D printing provides the possibility of creating, multiplying, adapting and sharing such data worldwide with little investment of recourses. Thus, an important contribution can be made for training this challenging and intimate examination. Sufficient data concerning the simulation of gynecological pelvic palpation is lacking. The scientific evaluation of this approach should be subject of future studies.

## Data Availability

The datasets used and analysed during the current study are available from the corresponding author on reasonable request.

## Abbreviations

ASTM: American Society for Testing and Materials
FDM: Fused Deposition Modeling
GTA: Gynaecology Teaching Associates
PETG: Polyethylene Terephthalate Glycol-modified
PLA: Polylactic Acid
SLA: Stereolithography
STL: Standard Tessellation Language
3D: Three Dimensional
2D: Two Dimensional
